# Supplementation of mixed *Lactobacillus* alleviates metabolic impairment, inflammation, and dysbiosis of the gut microbiota in an obese mouse model

**DOI:** 10.3389/fnut.2025.1554996

**Published:** 2025-03-26

**Authors:** Shulin Hou, Ruining Li, Yunyun Zhang, Ping Liang, Haishan Yang, Huili He, Lei Wang, Yaojun Sun, Tianru Jin, Zhizhen Liu, Jun Xie

**Affiliations:** ^1^Shanxi Key Laboratory of Birth Defect and Cell Regeneration, MOE Key Laboratory of Coal Environmental Pathogenicity and Prevention, Department of Biochemistry and Molecular Biology, Shanxi Medical University, Taiyuan, China; ^2^Academy of Medical Sciences, Shanxi Medical University, Taiyuan, China; ^3^Department of Biochemistry and Molecular Biology, College of Basic Medical Sciences, Shanxi Medical University, Taiyuan, China; ^4^Department of Physiology, Temerty Faculty of Medicine, Banting and Best Diabetes Centre, University of Toronto, Toronto, ON, Canada

**Keywords:** obesity, probiotics, high-fat-diet, anti-inflammation, lipid metabolism, gut microbiota

## Abstract

**Introduction:**

Obesity is a complex metabolic disease, which is often accompanied with impaired glucose and lipid metabolism and chronic inflammation. Probiotics have been considered as a strategy for treating obesity, while the genus of *Lactobacillus* is the most commonly tested and approved probiotics. Some multi-strain probiotics were proven to produce synergistic effects on treating obesity as compared to mono-strain ones.

**Methods:**

The purpose of this study was to investigate the anti-obesity effect of a new probiotic formation contained *Lactobacillus plantarum* L14, *Lactobacillus paracasei* L9, *Lactobacillus rhamnosus* GG, and *Lactobacillus sakei* X-MRS-2, designated as L-PPRS. Multi-strain probiotics L-PPRS was shown to have a better antiadipogenic effect than mono-strain probiotics in 3T3-L1 cell. Subsequently, L-PPRS was orally supplemented to a high-fat diet (HFD) induced obese mouse model for two kinds of treatment course, a short-term (8 weeks) one and a long-term (12 weeks) one.

**Results:**

We found that intervention of L-PPRS not only significantly inhibited weight gain in HFD-fed mice, but also improved glucose tolerance, insulin sensitivity and reduced serum lipid levels. Furthermore, L-PPRS intervention reduced fat accumulation in the adipose tissue and the liver, and ameliorated the antioxidant capacity of liver in HFD-fed mice. L-PPRS intervention modulated the expression of lipid-metabolic genes, and exhibited excellent anti-inflammatory effect. In addition, L-PPRS intervention restored the dysbiosis of gut microbiota via reducing the *Firmicutes/ Bacteroidetes* ratio, and increasing the abundance of beneficial intestinal bacteria. In conclusion, this study proved that L-PPRS could effectively prevent the development of obesity and its associated abnormalities, and the long-term supplementation of L-PPRS provided a more profound benefit than the short-term.

**Discussion:**

This study highlights the potential of L-PPRS as an effective anti-obesity strategy.

## 1 Introduction

Obesity is a complex metabolic disease defined as excessive adiposity (1). It is often associated with other chronic metabolic syndromes, such as nonalcoholic fatty liver disease, hypertension, type 2 diabetes, atherosclerosis and sleep apnoea, and has become an important global concern (2). Approximately 800 million people worldwide are living with obesity, and childhood obesity is expected to increase by 60% over the next decade, revealing the urgent and imperative response to prevent this global epidemic (3–5).

Obesity is intrinsically characterized by gut microbiota dysbiosis, inflammation and dysregulated glucose-lipid metabolism (6, 7), mostly triggered by the overconsumption of Western diet. Diet-microbiota interactions play an irreplaceable role in regulating gluconeogenesis and lipid biosynthesis, and are crucial moderators for the development of obesity (8). Considerable evidences suggest that obesity is associated with reduced diversity and abundance of the gut microbiota (9–11), and also with reduced *Firmicutes/ Bacteroidetes* (F/B) ratio (12–14). Long-term intake of high fat diet prevents the biosynthesis of beneficial metabolites in the gut, especially short chain fatty acids, then inhibits the enrichment of butyrate-producing bacteria, leading to glucose-lipid metabolism disorders, and even obesity (15, 16). To clear fat accumulation in adipose tissue, macrophages are recruited, releasing pro-inflammatory cytokines, which could induce systemic chronic inflammation and cause long-term and persistent damage of the body (17, 18). Adipose tissue macrophages also regulate obesity-related metabolic disorders, for instance, pro-inflammatory cytokines IL-1β, secreted from adipose tissue macrophages, is proved to be an important factor for the development of insulin resistance (19). Hence, given the strong association between gut microbiota dysbiosis, inflammation, metabolism and obesity, an effective anti-obesity strategy should not only simply inhibit body weight gain, but also modulate abnormalities inside.

Various approaches are currently employed in the treatment of obesity, including lifestyle interventions, pharmacotherapy, and surgical interventions (20). Lifestyle intervention attenuates obesity through healthy eating and physical activities, which is difficult to persist and may not bring long-term benefits (21, 22). Less than 50% of the patients were able to achieve a weight loss of 5% or more, and there is a potential for rebound (23). Pharmacotherapy represents an advanced intensive treatment modality (24). Several medications are currently approved for obesity treatment, such as orlistat, phentermine and liraglutide (25–27). These drugs may achieve the expected results, however most of them may produce gastrointestinal side effects and adverse reactions (28). Common bariatric surgeries include gastric banding, sleeve gastrectomy, Roux-en-Y gastric bypass, and biliopancreatic diversion (29), these procedures are invasive and entail a higher treatment cost (30). Therefore, a more effective and safer approach to treating obesity is required.

Probiotics have been highlighted as a new strategy in the prevention and treatment of metabolic disorders (31, 32), and the genus of *Lactobacillus* are the most commonly tested probiotics for treating obesity (33). *Lactobacillus rhamnosus* GG (*L. rhamnosus* GG) may alleviate obesity via attenuating leptin resistance and decreasing the proportion of Proteobacteria in fecal microbiota (34). *Lactobacillus plantarum* L14 (*L. plantarum* L14) was shown to inhibit fat formation, thereby alleviating systemic inflammation and reducing obesity (35, 36). *Lactobacillus paracasei* L9 (*L. paracasei* L9) could ameliorate HFD-induced lipid accumulation and inflammation associated with gut dysbiosis in mice (37). *Lactobacillus sakei* (*L. sakei*) was shown to play a significant role in the fermentation process of Kimchi (38). A few studies have demonstrated that *L. sakei* induced anti-obesity effect by restoring F/B ratio and enhancing the abundance of strains for butyrate production (39, 40). Besides *L. sakei*, the genus of *Lactobacillus* are the most commonly tested probiotics for modifying F/B ratio (6, 33). Furthermore, obesity-related lipid levels have strong negative correlation with the abundance of *Lactobacillus* (41). In addition, *Lactobacillus* intervention reshaped and increased the diversity of the gut microbiome, especially increasing the abundance of *Akkermansia* (42, 43), which was regarded as the paradigm for next-generation beneficial gut microbiomes (44). In a word, numerous strains of *Lactobacillus* have been shown to be effective in anti-obesity via diverse mechanisms.

Probiotics contained multi-strains, in great quantity on the market, were reported to be safe and effective for obesity treatment (45–47). Compared with the single-strain probiotics, multi-strain probiotics achieved better therapeutic effect by enhancing intestinal colonization ability (48) and producing synergistic effect (49). As mentioned above, several *Lactobacillus* spp. including *L. plantarum* L14 (L14), *L. paracasei* L9 (L9), *L. rhamnosus* GG (LGG), and *L. sakei* X-MRS-2 (L-X-MRS-2), present anti-obesity effect, potentially with different mechanisms. In our current study, we have investigated the effect of these mixed *Lactobacillus* (L-PPRS) on anti-obesity and explored related mechanism. We have firstly verified a better antiadipogenic capacity of L-PPRS than mono-strain probiotics in 3T3-L1 cell. To further analyze the anti-obesity effect of L-PPRS and related mechanism, L-PPRS was orally supplemented to an HFD induced obese mouse model for two kinds of treatment course, a short-term one (8 weeks) and a long-term (12 weeks) one. The obesity-related indicators including weight gain, plasma lipid levels, insulin tolerance, insulin resistance, fat accumulation, liver function, antioxidant capacity of liver, and inflammatory state were determined. To further study the anti-obesity mechanism of L-PPRS, the expression of lipid metabolism-related genes, and the modulation in gut microbiota dysbiosis were analyzed.

## 2 Materials and methods

### 2.1 Preparation of compound probiotics L-PPRS

*L. plantarum* L14 (L14), *L. paracasei* L9 (L9), *L. rhamnosus* GG (LGG), and *L. sakei* X-MRS-2 (L-X-MRS-2) used in this experiment were purchased from the CHINA CENTER OF INDUSTRIAL CULTURE COLLECTION (CICC) (Shanghai, China). Strains were cultured in Man Rogosa Sharpe (MRS) broth at an appropriate temperature (L14 is at 30°C, L9, LGG and L-X-MRS-2 are at 37°C) for 24 h, and then they were centrifuged (4,200 rpm, 10 min, 4°C) to collect bacterial precipitation. After washing twice with PBS, cells were suspended in PBS at the concentration of 1×10^9^ colony forming units (CFU) /mL. The four strains were mixed according to 1:1:1:1 ratio to form the compound probiotics (L-PPRS).

### 2.2 Cell culture

The mouse preadipocyte cell line 3T3-L1 was purchased from Wuhan Pricella Biotechnology Co, Ltd. (Wuhan, China). Cells were cultured in Dulbecco’s modified Eagle’s medium (DMEM) (Gibco, America) supplemented with 10% (v/v) Newborn Calf Serum (NCS) and 1% (v/v) antibiotic solution (penicillin and streptomycin) in a humidified environment containing 5% CO_2_ at 37°C. For adipogenic differentiation, cells were seeded at a density of 1×10^5^ cells/well in a 12-well cell culture plates and grown to 100% confluence in the maintenance medium (DMEM containing 10% NCS and 1% antibiotic solution). Two days after full confluency (referred as day 0), the maintenance medium was replaced by induction medium [DMEM supplemented with 10% (v/v) FBS, 1% antibiotic solution, 0.5 mM 3-isobutyl-1-methylxanthine (IBMX) (Solarbio), 1 μM dexamethasone (Solarbio) and 10 μg/mL of insulin (Solarbio)]. After 48 h, the medium was switched to induction medium only containing 10 μg/mL of insulin. The induction medium containing insulin was changed every 2 days to induce differentiation for 8 days.

Before the differentiation of cells, heat-inactivated (95°C, 15 min) probiotic strains resuspended in PBS were added and incubated with the cells for 24 h and 48 h. These cells were collected for the determination of cytotoxic effects.

During the 10 days induction of differentiation, heat-inactivated strains were added at 2% volume of the total medium, and changed every 2 days. These cells were collected for oil red O Staining.

### 2.3 Cell viability (MTT)

The cytotoxic effects were determined by MTT assay. Mouse 3T3-L1 cells were inoculated at 5,000 cells/ well in a 96-well plate for 24 h. Then heat-inactivated L14, L9, LGG, L-X-MRS-2 and L-PPRS at a concentration of 1×10^9^ CFU/mL were added to the medium at 2, 4, 6, 8 and 10% (v/v) volume of the total medium, respectively, and incubated for 24 h and 48 h. After discarding the supernatant, 90 μL of culture medium and 10 μL of MTT solution (Solarbio) were added to each well. After incubation at 37°C with 5% CO_2_ for 4 h, the supernatant was aspirated and 110 μL of formazan solvent (Solarbio) was added to each well. The plate was shaken at a low speed for 10 min to fully dissolve the crystalline material. The absorbance of each well was measured at 490 nm using a microplate reader (Molecular Devices, America).

### 2.4 Oil red O staining

After 10 days induction of differentiation, the cell monolayer was washed twice with PBS and fixed with 4% paraformaldehyde (Beyotime, Shanghai, China) for 10 min. After washing twice with PBS again, cells were stained with modified oil red O staining solution (Beyotime) for 20 min, and then washed with staining washing solution and PBS. Images of the stained cells were obtained using a microscope Ti2 (Nikon, Japan). To quantify lipids in cells, oil red O-stained lipid droplets were dissolved with 100% isopropanol for 10 min with gentle shaking. The absorbance of the eluted solution was measured at 500 nm using 100% isopropanol as a blank with a microplate reader (Molecular Devices, America).

### 2.5 Animals and experimental design

The animal experiments were conducted in full compliance with the guidelines of the Animal Ethics Committee of Shanxi Medical University (approval no. 2024-113). All authors were aware of the assignment of groups in the different phases of the experiment.

To conduct this study, the inclusion criteria were defined as follows: male C57BL/6 mice aged 4-weeks and weighing 15–20 g; any mice not meeting these parameters at the start of the study were excluded. C57BL/6 mice (4 weeks old) were purchased from the Animal Experiment Center of Shanxi Medical University, and acclimatized for 1 week. Animals were housed in cages with 3–4 mice per cage under a 12-h light–dark cycle at 22°C for a duration of 12 weeks. Based on previous literature regarding the efficacy of probiotics in attenuating obesity (50, 51), the sample size of 6 animals per group was determined.

After 1-week acclimatization, 24 mice were randomized into 4 groups: (1) normal diet (ND) (*n* = 6), (2) high fat diet (HFD) (*n* = 6), (3) HFD + short-term L-PPRS (HFD-CP1, 8 weeks) (*n* = 6), and (4) HFD + long-term L-PPRS (HFD-CP2, 12 weeks) (*n* = 6). HFD group, HFD-CP1 group and HFD-CP2 group were fed with D12492 high-fat feed during 1–12 weeks, which was manufactured by BEIJING BOAIGANG BIOLOGICAL TECHNOLOGY CO., LTD (Beijing, China). Mice in the HFD-CP1 group were gavaged with 0.2 mL of sterile PBS during 1–4 weeks and 0.2 mL of L-PPRS at a concentration of 1×10^9^ CFU/mL during 5–12 weeks. Mice in the HFD-CP2 group were given with 0.2 mL of L-PPRS at a concentration of 1×10^9^ CFU/mL for 1–12 weeks. Mice in the ND and HFD groups were gavaged with 0.2 mL of sterile PBS for 12 weeks. During the experimental period, mice had free access to water and food. The body length and weight of each mouse were recorded every week. Lee′ index was calculated as follows:


$${Lee}^{\prime }\;s\;\mathrm{index}=\sqrt[{3}]{\mathrm{body weight}\;\left(g\right)}\times 10÷\mathrm{body length}\;\left(\mathrm{cm}\right)\text{.}$$

### 2.6 Glucose and insulin tolerance tests

The oral glucose tolerance test (OGTT) was performed after fasting for 12 h. Each mouse was gavaged with glucose (2 mg/g body weight). Blood samples were collected from mouse tail vein for blood glucose level determination at 0, 15, 30, 60, 90, and 120 min following oral glucose gavage, and the area of the curve (AOC) was calculated. The insulin tolerance test (ITT) was performed after fasting for 6 h. Each mouse was injected with insulin (0.75 U/kg body weight), and blood glucose levels were measured at 0, 15, 30, 60, 90, and 120 min following injection, and the area of the curve (AOC) was calculated.

### 2.7 Biochemical analysis

Blood was collected and allowed to stand at 25°C for 1 h, and centrifuged (3,000 g, 10 min, 4°C) to obtain serum. A 10% liver homogenate was prepared by combining the liver (g) with saline (mL) in 1:9 ratio, and the supernatant was collected by centrifugation (4°C, 8,000 rpm, 10 min). The serum lipid levels of total cholesterol (TC), triglyceride (TG), low-density lipoprotein cholesterol (LDL-C), high-density lipoprotein cholesterol (HDL-C), and non-esterified Free fatty (NEFAs) were assayed using commercial kits according to the manufacturer’s instructions (Nanjing Jiancheng Bioengineering Institute). The levels of TC, TG, NEFAs, aminotransferase (ALT), aspartate aminotransferase (AST) in the livers were also detected using commercial assay kits (Nanjing Jiancheng Bioengineering Institute). The levels of malondialdehyde (MDA) and glutathione (GSH) (Nanjing Jiancheng Bioengineering Institute), as well as the activities of Superoxide dismutase (SOD) (Beyotime Biotechnology, China) and catalase (CAT) (Solarbio) in the livers were determined using the commercial kits according to the manufacturer’s guidelines, respectively. Levels of inflammatory cytokines TNF-α, IL-1β, IL-6, and IL-10 in mouse serum were measured using correspondent commercial ELISA kits (Solarbio).

### 2.8 Histological analysis

Mouse liver and epididymal adipose tissue were immersed in 4% paraformaldehyde for fixation. After dehydration, tissues were paraffin-embedded and cut into sections. Sections were stained with hematoxylin and eosin (H&E) for further histopathological analysis. These results were photographed using a microscope Ti2 (Nikon).

### 2.9 RT-qPCR analysis

Total RNA from epididymal adipose tissue was extracted using an RNA extraction kit (TransGen Biotech, Beijing, China). cDNA was obtained using a reverse transcription kit (Abm, Canada, Inc.). qPCR analysis was conducted using the abm BlasTaq^™^ 2X qPCR MasterMix and primers. Primer sequences for genes related to lipid metabolism are shown in Supplementary Table S1, including those that encode fatty acid synthase (FAS), acetyl CoA carboxylase (ACC), sterol regulatory element-binding protein-1 (SREBP-1C), Adenosine 5′-monophosphate (AMP)-activated protein kinase α (AMPK-α), Carnitine palmitoyltransferase1 (CPT-1), peroxisome proliferator-activated receptor (PPAR-γ), CCAAT enhancer binding protein α (CEBP-α) and hormone-sensitive triglyceride lipase (HSL).

### 2.10 Microbial analysis of cecal contents

Microbiota of mouse cecal contents were analyzed by 16S rDNA sequencing. After dissection, the cecal contents of mice were collected into a freezing tube, which was immediately placed in liquid nitrogen and transferred to −80°C for storage. Total genome DNA was extracted using E.Z.N.ATM.Mag-Bind Soil DNA kit (Omega, M5635-02, United States), and the concentration of DNA was measured using Qubit 4.0 (Thermo, United States) to ensure the adequate amount of high-quality genomic DNA. The V3–V4 amplicon of 16S rRNA was amplified using 2 × Hieff® Robust PCR Master Mix (Yeasen, 10105ES03, China). Two universal bacterial 16S rRNA gene amplicon PCR primers (PAGE purified) were used: the amplicon PCR forward primer (CCTACGGGNGGCWGCAG) and amplicon PCR reverse primer (GACTACHVGGGTATCTAATCC). The plate was sealed and PCR performed in a thermal instrument (Applied Biosystems 9700, United States). Hieff NGS^™^ DNA Selection Beads (Yeasen, 10105ES03, China) was used to purify PCR products. Samples were delivered to Sangon BioTech (Shanghai, China) for library construction and analysis using universal Illumina adaptor and indices. Sequences were analyzed by PEAR software (version 0.9.8), the effective tags were clustered into operational taxonomic units (OTUs) of ≥97% similarity using Usearch software (version 11.0.667). The α-diversity indices were quantified in terms of OTU richness with Mothur software (version 3.8.31). β-diversity were visualized using R vegan package (version 2.5–6). Difference comparison is used to identify features with significantly different abundances between groups using STAMP (version 2.1.3) and LefSe (version 1.1.0).

### 2.11 Statistical analysis

Experimental data were expressed as mean ± standard deviation (Mean ± SD). Software GraphPad Prism 8.0 was used for statistical analysis. For comparisons involving two or more groups, a one-way analysis of variance (ANOVA) was performed, followed by multiple comparisons using the *t*-test to determine significant differences. *p* values of less than 0.05 were considered statistically significant.

## 3 Results

### 3.1 Effect of L-PPRS on antiadipogenic capacities of 3T3-L1 cells

**Figure 1:**
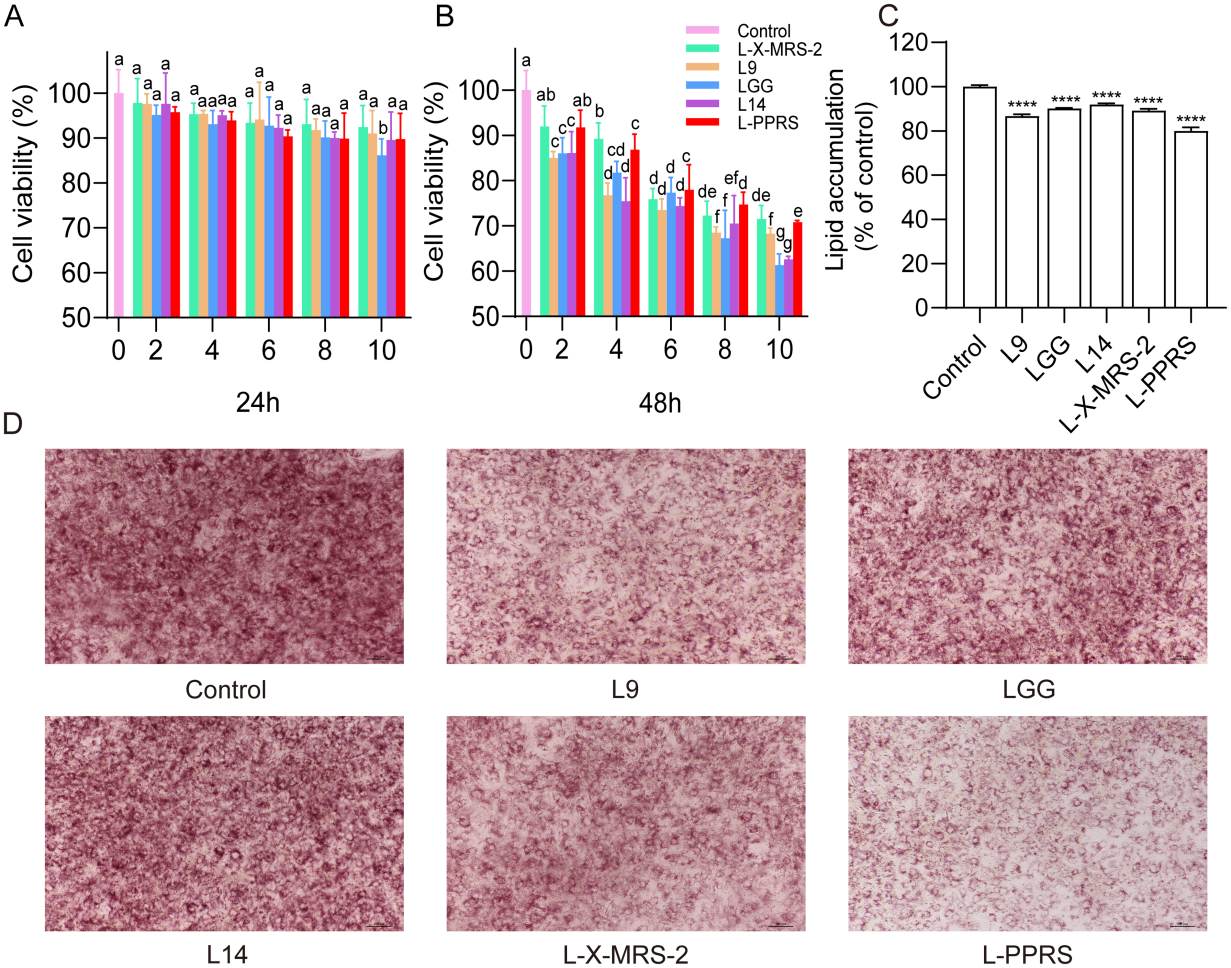
Effects of probiotics on lipid accumulation in 3T3-L1 adipocytes. **(A)** Cell viability (24 h). **(B)** Cell viability (48 h). Different letters in a column indicate significant difference (*p* < 0.05). **(C)** Relative level of lipid content in 3T3-L1 cells. **(D)** Oil red O staining (The ratio of the lipid droplet area to the total area is presented in Supplementary Figure S1). Results are expressed as mean ± SD (*n* = 3) (****p < 0.0001 vs. control).

3T3-L1 adipocytes were employed to assess the effect of probiotics on cytotoxicity and lipid accumulation. Firstly, MTT assay was used to evaluate the potential cytotoxicity effect in 3T3-L1 cells. Four kinds of single strain probiotics including L14, L9, LGG, and L-X-MRS-2, and their mixed strains L-PPRS, were heat-inactivated, and then incubated with 3T3-L1 cells for 24 h and 48 h (Figures 1A,B). The results showed that 3T3-L1 cells, which treated with probiotics for 24 h, maintained good viability (>85%) in all groups treated with either four kinds of single strain probiotics or mixed strains L-PPRS at 2–10% concentrations (Figure 1A). When incubation for 48 h, good cell viability (>85%) was maintained only in groups treated with 2% concentration of probiotics (Figure 1B). Therefore, 2% concentration was chosen as the experimental concentration for the further study.

To determine their potential antiadipogenic effect, 3T3-L1 cells were treated with heat-inactivated probiotics for 10 days, and then stained with oil red O to observe lipid droplet accumulation, lipids in cells were further quantified. Results showed that all probiotic strains, especially L-PPRS, suppressed lipid accumulation in 3T3-L1 cells, when compared with the control group (Figures 1C,D). Thus, these *Lactobacillus* probiotics have the potential to anti-obesity by suppressing lipid accumulation, and mixed *lactobacillus* strains L-PPRS is more effective than single-strain probiotics.

### 3.2 Effect of L-PPRS on growth performance and metabolic profile in HFD challenged mice

**Figure 2:**
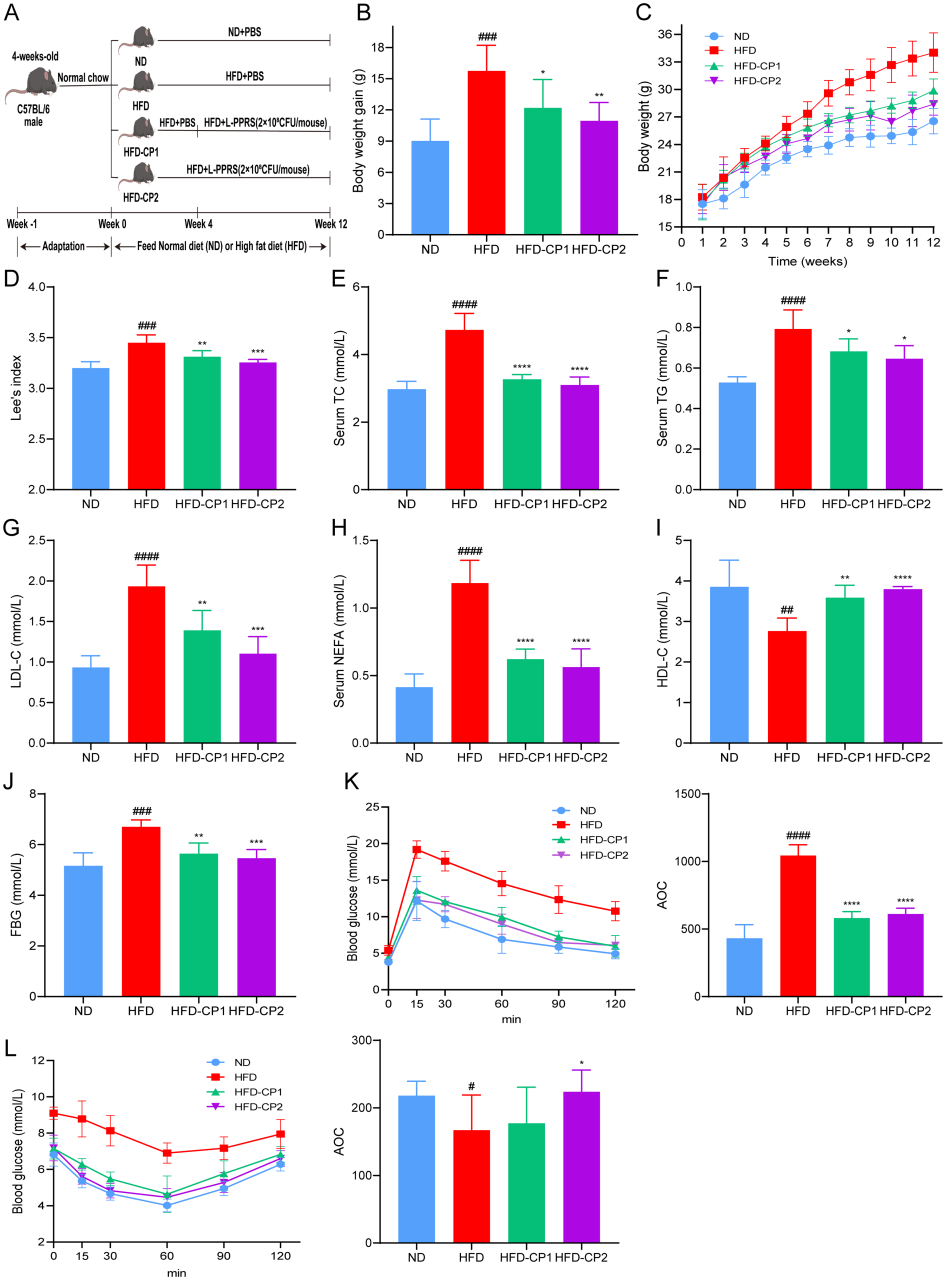
Effects of L-PPRS intervention on body weight gain, glucose tolerance and insulin resistance. **(A)** Schematic presentation of animal experiment. **(B)** Body weight gain. **(C)** Body weight. **(D)** Lee’s index. **(E)** TC. **(F)** TG. **(G)** LDL-C. **(H)** NEFA. **(I)** HDL-C. **(J)** Fasting blood glucose levels. **(K)** Plasma glucose level during mouse glucose tolerance test. AOC quantification of OGTT results (right). **(L)** Plasma glucose level during GTT. AOC quantification of GTT results (right). All data are expressed as the mean ± SD (*n* = 6) (^#^*p* < 0.05, ^##^*p* < 0.01, ^###^*p* < 0.001, ^####^*p* < 0.0001 vs. ND; *p < 0.05, **p < 0.01, ***p < 0.001, ****p < 0.0001 vs. HFD).

The anti-obesity effect of L-PPRS was evaluated in terms of body weight and Lee’s index in HFD induced obese mice, designed with two kinds of treatment course (Figure 2A), a short-term HFD-CP1 (8 weeks) course and a long-term HFD-CP2 (12 weeks) course. At the end of 12 weeks, the body weight gain of the HFD group (Final weight, 34.01 g ± 3.22 g) was 28% (>20%) higher than that of ND group (26.54 g ± 2.52 g) (Figure 2B), indicating that the obese model was successfully established. After L-PPRS intervention, the weight gain of mice decreased significantly with 22.63 and 30.49% in HFD-CP1 group and HFD-CP2 group (Figure 2B), respectively, showing the correlation between increased intervention period and anti-obesity effect of L-PPRS. As shown in Figure 2C, the growth rate of the HFD-CP2 group was slower than that of the HFD and HFD-CP1 groups, while the rate of the HFD-CP1 group was close to the HFD group at 1–4 week, and began to slow down after L-PPRS intervention at week 5. In addition, Lee’s index decreased significantly in response to L-PPRS intervention (Figure 2D).

Obesity is often accompanied by abnormal serum lipid levels, including TC, TG, LDL-C, HDL-C, and NEFAs. In our current study, after L-PPRS intervention to HFD challenged mice, HFD-CP1 and HFD-CP2 group had decreases of 30.89 and 34.44% in TC (Figure 2E), 13.89 and 18.55% in TG (Figure 2F), 28.12 and 42.97% in LDL-C (Figure 2G), 47.56 and 52.49% in NEFAS (Figure 2H), and had increases of 29.81 and 37.37% in HDL-C (Figure 2I), separately. Compared with the HFD-CP1 group, the long period supplementation of L-PPRS (HFD-CP2) significantly decreased LDL-C level and increased HDL-C level. Collectively, L-PPRS could effectively alleviate the weight gain and the lipid level in obese mice.

HFD feeding can also lead to abnormality in glucose homeostasis. At 12 weeks, fasting blood glucose level in the HFD group was 6.7 mmol/L (Figure 2J), higher than that in the ND group (5.16 mmol/ L). During OGTT performance, the glucose levels in the HFD group reached 14.54 and 10.76 mmol/L after 60 min and 120 min, respectively, indicating the development of impaired glucose tolerance (Figure 2K). After L-PPRS intervention, glucose levels of HFD mice were decreased to 9.96 and 8.98 mmol /L after 60 min in HFD-CP1 and HFD-CP2 group, and both returned to the normal level (<7.8 mmol/L) after 120 min. During insulin ITT performance (Figure 2L), after L-PPRS intervention, especially in the HFD-CP2 group, the mice exhibited significantly decreased blood glucose levels, indicating the improvement of insulin sensitivity, as evidenced by the increase inverse Area of the Curve (AOC) of ITT. These results indicate that the L-PPRS intervention effectively improved glucose tolerance and insulin sensitivity.

In a word, our data indicates that L-PPRS supplementation can alleviate the weight gain, maintain the serum lipid level, and improve glucose tolerance and insulin sensitivity in HFD challenged mice.

### 3.3 L-PPRS intervention reduces fat accumulation in the adipose tissue in HFD challenged mice

**Figure 3:**
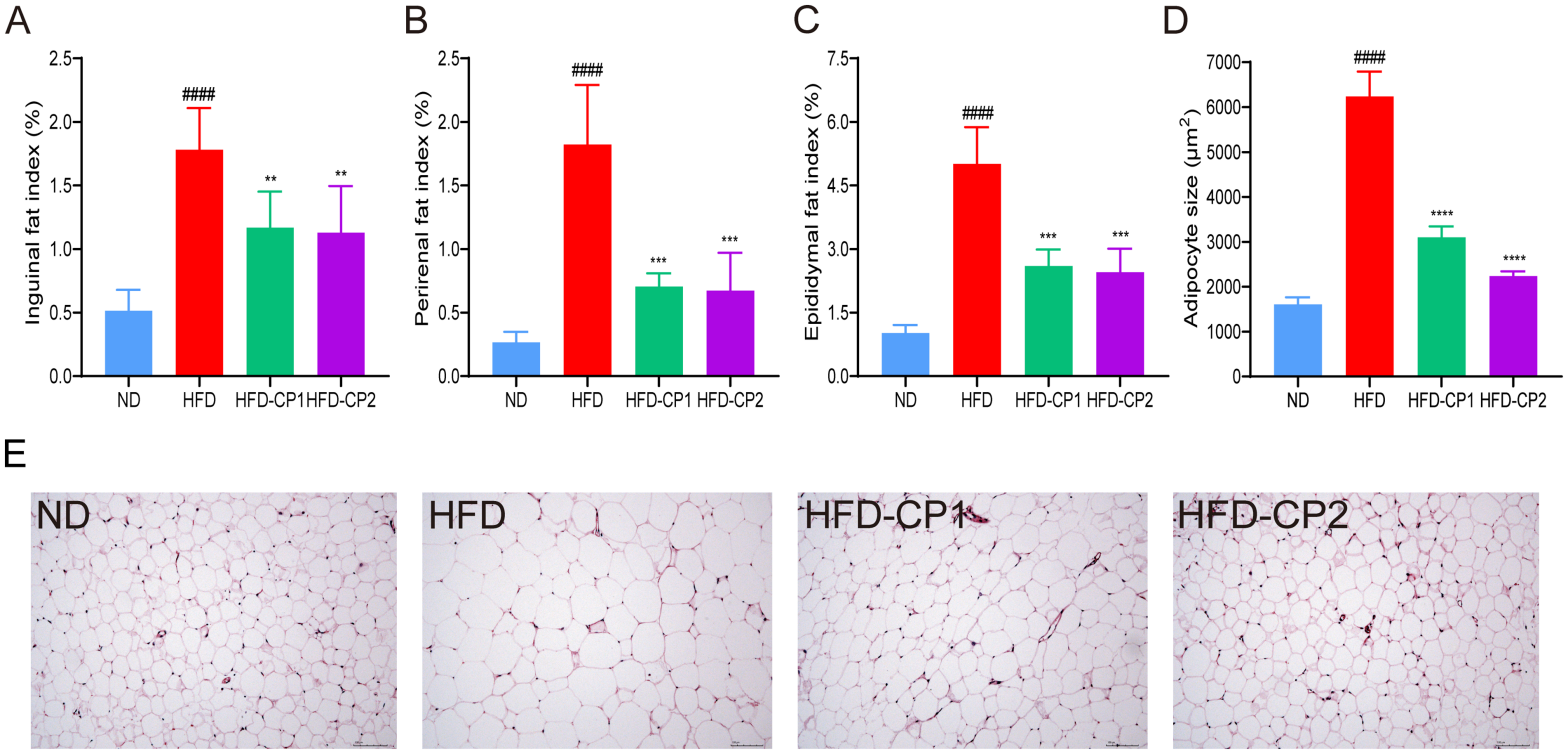
Effects of L-PPRS intervention on fat accumulation in mice. **(A)** Inguinal fat index. **(B)** Perirenal fat index. **(C)** Epididymal fat index. **(D)** Mean of adipocyte size. **(E)** Adipose tissue H&E staining. All data are expressed as the mean ± SD (*n* = 6) (^####^*p* < 0.0001 vs. ND; **p < 0.01, ***p < 0.001 vs. HFD).

Excessive fat accumulation in adipose and other tissues is one of the main characteristics of obesity. As shown in Figures 3A–C, the fat index in groin (Figure 3A), kidney (Figure 3B), and epididymis (Figure 3C) of the HFD group increased 2.46, 5.86, and 3.92 times, respectively, compared to the ND group. After L-PPRS intervention, the above fat indexes in HFD-CP1 and HFD-CP2 group decreased significantly compared with HFD group. Quantitative analysis of adipocyte size confirmed that L-PPRS treatment in HFD-CP1 and HFD-CP2 groups demonstrated a significant decrease in adipocyte volume of the epididymal fat tissue (Figure 3D). HE staining on epididymal fat of mice was performed to further evaluate the morphological changes of adipose tissue. As shown in Figure 3E, adipocytes in the ND group displayed a small size and neatly arrangement. In contrast, the HFD group exhibited hypertrophic and inhomogeneous adipocytes. The fat cells became uniformed and orderly arranged after L-PPRS intervention. Compared with HFD-CP1 group, the HFD-CP2 group had more uniformed adipocytes, similar to the ND group. These results suggest that L-PPRS significantly reduces the fat accumulation in adipose tissue of HFD challenged mice.

### 3.4 L-PPRS intervention reduces hepatic fat accumulation and ameliorates liver damages

**Figure 4:**
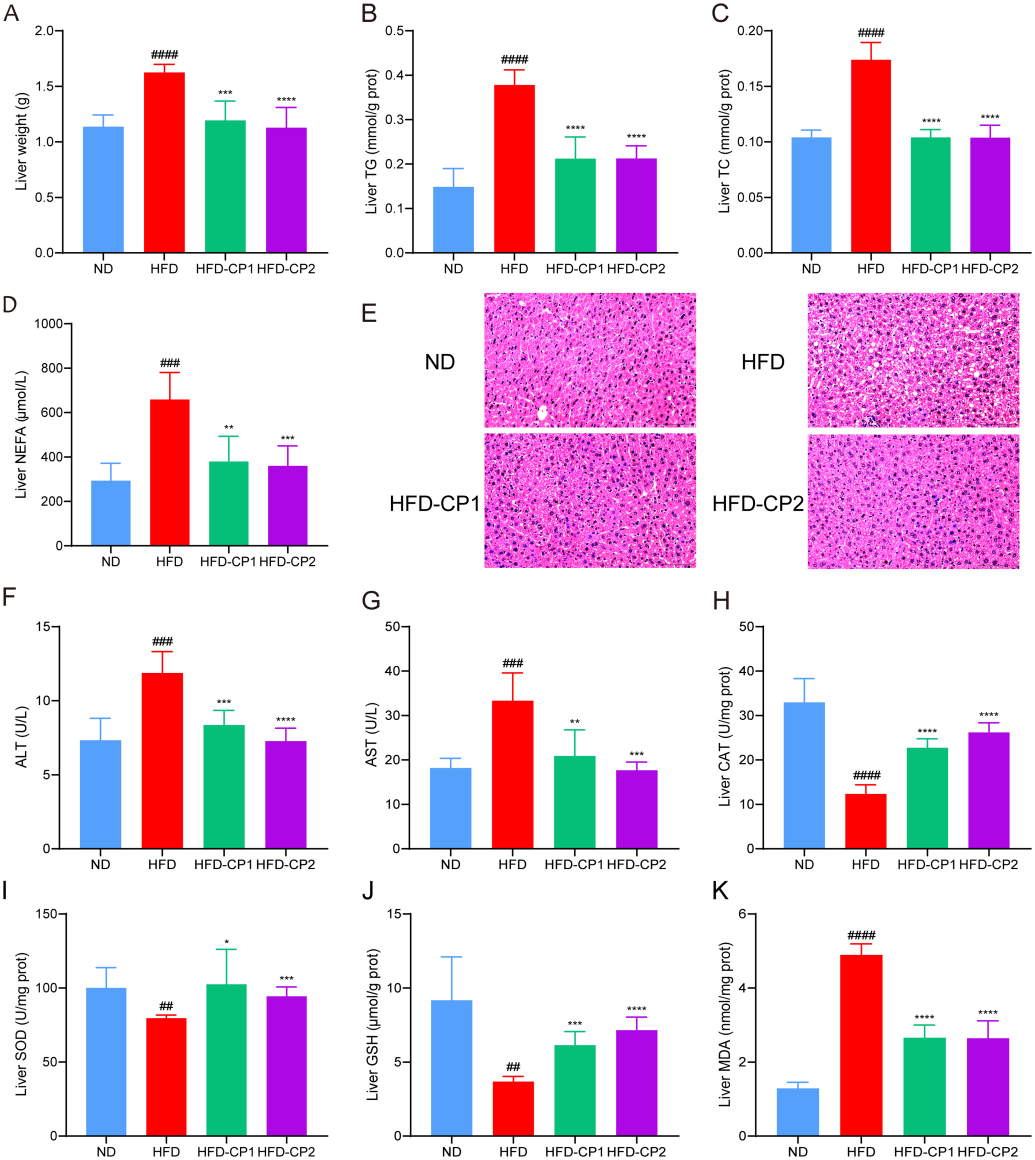
Effects of L-PPRS on lipid levels and antioxidant indexes in the liver. **(A)** Liver weight. **(B)** Liver TG. **(C)** TC. **(D)** NEFA. **(E)** Liver pathological injury **(F)** ALT. **(G)** AST. **(H)** CAT. **(I)** SOD. **(J)** GSH. **(K)** MDA. All data are expressed as the mean ± SD (*n* = 6) (^#^*p* < 0.05, ^##^*p* < 0.01, ^###^*p* < 0.001, ^####^*p* < 0.0001 vs. ND; *p < 0.05, **p < 0.01, ***p < 0.001, ****p < 0.0001 vs. HFD).

Except for adipose tissues, HFD challenge can also led to fat accumulation in the liver, impairing hepatic functions. As shown in Figure 4A, HFD challenge significantly increased liver weight (42.96%). More specifically, TG (Figure 4B), TC (Figure 4C), and NEFAs (Figure 4D) levels in the livers of HFD mice increased 1.54, 0.67, and 1.24 times, separately, compared with the ND group. In contrast, after L-PPRS supplementation to HFD challenged mice, HFD-CP1 and HFD-CP2 group had decreases of 26.56 and 30.56% in liver weight, 43.90 and 43.81% in TG, 40.18 and 40.31% in TC, 42.43 and 45.39% in NEFAs, respectively, compared to HFD group. Meanwhile, HE staining of mouse liver tissues showed obvious hepatic steatosis with accumulated lipid droplets and internal fat cavities in the HFD group (Figure 4E). Conversely, after L-PPRS treatment, especially in HFD-CP2 group, hepatocytes are neatly arranged without fatty degeneration, similar to those in the ND group (Figure 4E, histological assessments were conducted solely through visual inspection without quantitative measurements to determine statistical significance). These results verify that L-PPRS treatment in HFD mice significantly reduces the accumulation of lipid in the liver compared to HFD group. Except liver, there were no significant differences in the organ indexes in high-fat diet (Supplementary Figure S2).

To further evaluate the liver function, we analyzed serum ALT and AST levels, and hepatic antioxidant levels (including CAT, SOD, GSH, and MDA levels). In the HFD group, serum levels of ALT (Figure 4F) and AST (Figure 4G) were increased 0.62 and 0.83 times separately, compared to those in the ND group, indicating that the liver was damaged. After L-PPRS intervention (in both HFD-CP1 and HFD-CP2 groups), the levels of ALT and AST were significantly reduced, and were closer to the normal level in the HFD-CP2 group. For the antioxidant status in the liver, HFD challenge decreased activities of antioxidant enzymes (CAT and SOD) (Figures 4H–I) and non-enzymatic antioxidant GSH (Figure 4J), leading to excess generation of peroxides (MDA) (Figure 4K). L-PPRS intervention significantly restored the antioxidant capacity of the liver, and in the long period intervention of L-PPRS (HFD-CP2) group, the mice showed slightly better SOD and GSH activities. In a word, L-PPRS could effectively resist the fat accumulation in liver and reduce liver damage in mice caused by HFD.

### 3.5 L-PPRS intervention affects the expression of lipid-metabolic genes in HFD challenged mice

**Figure 5:**
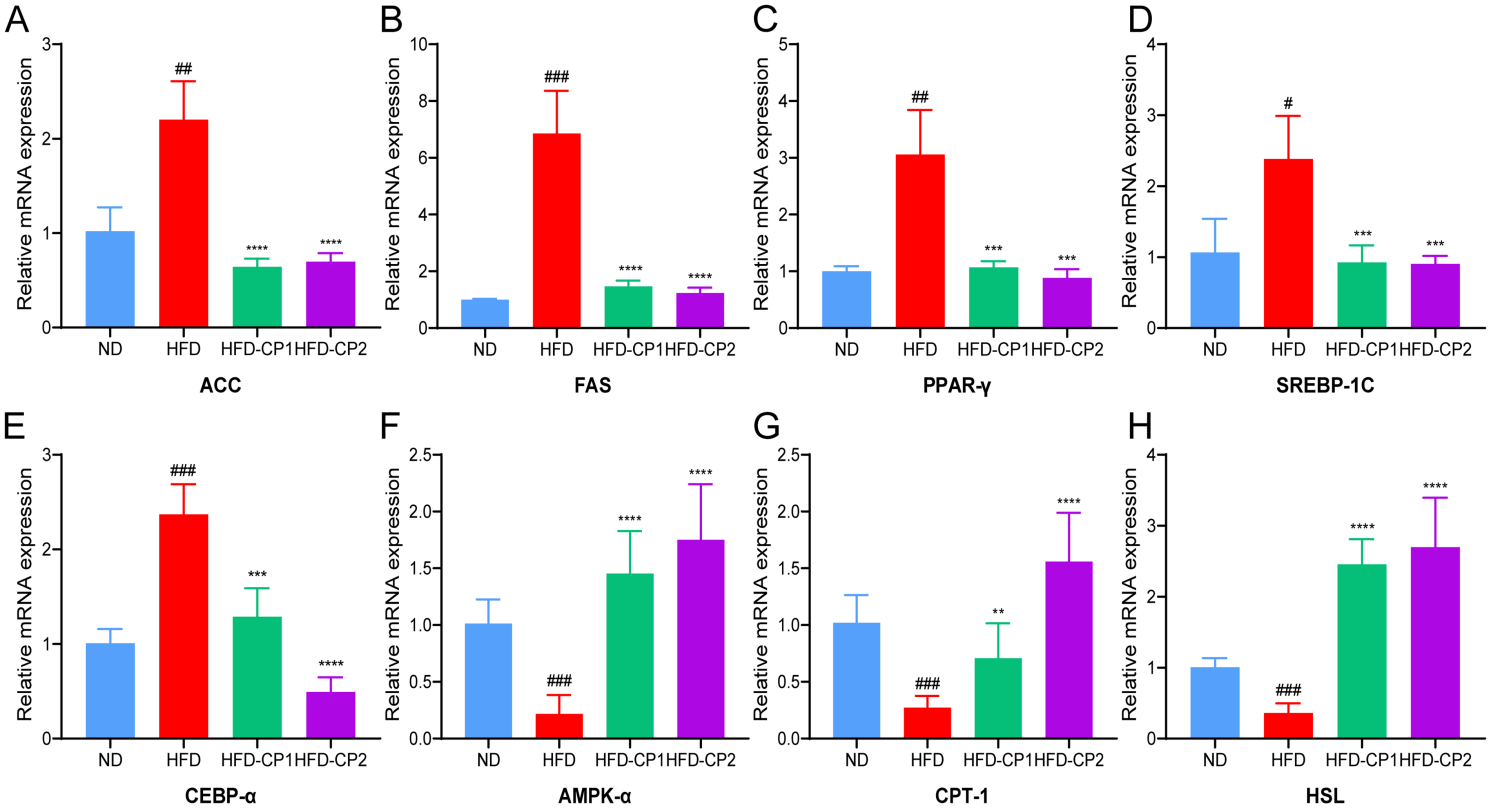
Effects of L-PPRS intervention on expression of genes that are related to lipid metabolism. **(A)** ACC. **(B)** FAS. **(C)** PPAR-γ. **(D)** SREBP-1C. **(E)** CEBP-α. **(F)** AMPK-α. **(G)** CPT-1. **(H)** HSL. All data are expressed as the mean ± SD (*n* = 6) (#*p* < 0.05, ##*p* < 0.01, ###*p* < 0.001 vs. ND; **p < 0.01, ***p < 0.001, ****p < 0.0001 vs. HFD).

In the adipose tissue of the HFD group, expression levels of lipid synthesis-related genes were significantly increased, including those that encode acetyl CoA carboxylase (ACC), fatty acid synthase (FAS), peroxisome proliferator-activated receptor (PPAR-γ), sterol regulatory element-binding protein-1 (SREBP-1C), and CCAAT enhancer binding protein α (CEBP-α) (Figures 5A–E), while expression levels of lipid lysis-related genes including those that encode Adenosine 5′-monophosphate (AMP)-activated protein kinase α (AMPK-α), Carnitine palmitoyltransferase1 (CPT-1), and hormone-sensitive triglyceride lipase (HSL) genes were significantly decreased (Figures 5F–H). L-PPRS intervention significantly reduced the expression levels of those that encode lipid synthesis-related genes including ACC, FAS, PPAR-γ, SREBP-1C, and CEBP-α, and increased the expression levels of those that encode lipid lysis-related genes including AMPK-α, CPT-1, and HSL. In addition, long period intervention had a more significant effect on regulating the gene expression of CEBP-α and CPT-1. Therefore, L-PPRS effectively regulates lipid metabolism in mice.

### 3.6 L-PPRS intervention attenuates HFD-induced systematic inflammation

**Figure 6:**
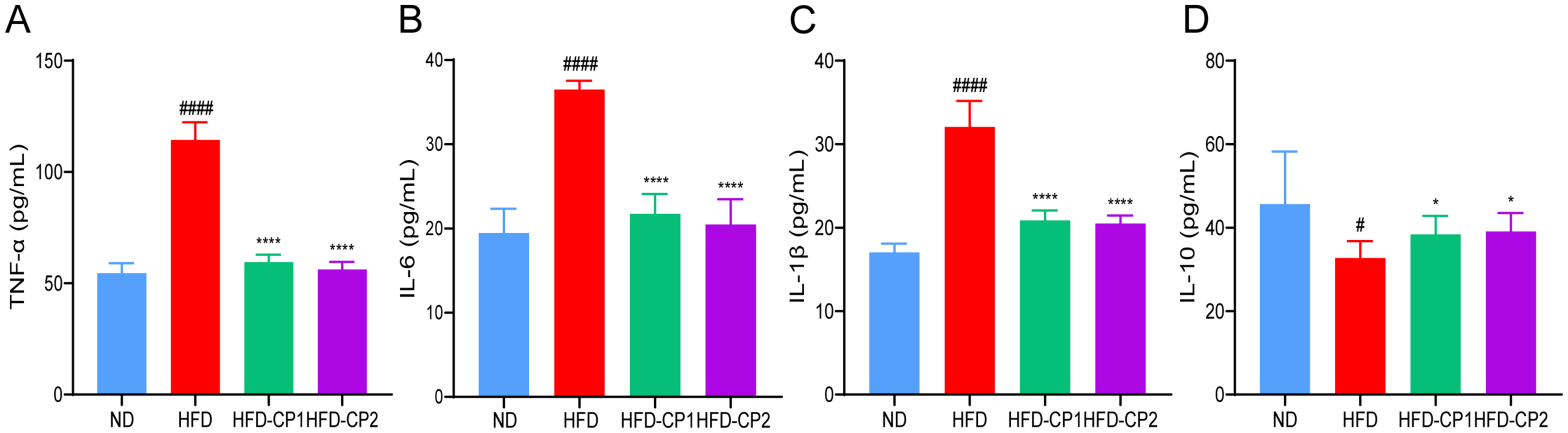
Effects of L-PPRS intervention on serum inflammatory factors in designated mouse groups. **(A)** TNF-α, **(B)** IL-6, **(C)** IL-1β. **(D)** IL-10. All data are expressed as the mean ± SD (*n* = 6) (^#^*p* < 0.05, ^####^*p* < 0.0001 vs. ND; *p < 0.05, ****p < 0.0001 vs. HFD).

Chronic inflammation is often associated with obesity and its related metabolic disorders. We hence evaluated the levels of pro-inflammatory factors including TNF-α, IL-1β, and IL-6 as well as the anti-inflammatory cytokine IL-10. As expected, HFD challenge significantly increased levels of TNF-α (Figure 6A), IL-1β (Figure 6B), and IL-6 (Figure 6C), and decreased the level of IL-10 (Figure 6D). Conversely, when HFD challenged mice supplemented with L-PPRS for 8 weeks, the mice displayed 47.92, 34.95, and 40.44% reduction in TNF-α, IL-1β, and IL-6, respectively; and 18.92% increase in IL10. These results indicate that L-PPRS intervention could significantly alleviate systematic inflammation in the HFD induced obese mice.

### 3.7 L-PPRS intervention prevents gut microbiota dysbiosis induced by HFD challenge

Diet-microbiota interaction is a crucial modulator in the development of obesity (8). Therefore, the effect of L-PPRS intervention on gut microbiota composition in HFD challenged mice were investigated by amplicon sequencing of the bacterial 16 s rRNA gene (the V3-V4 region).

**Figure 7:**
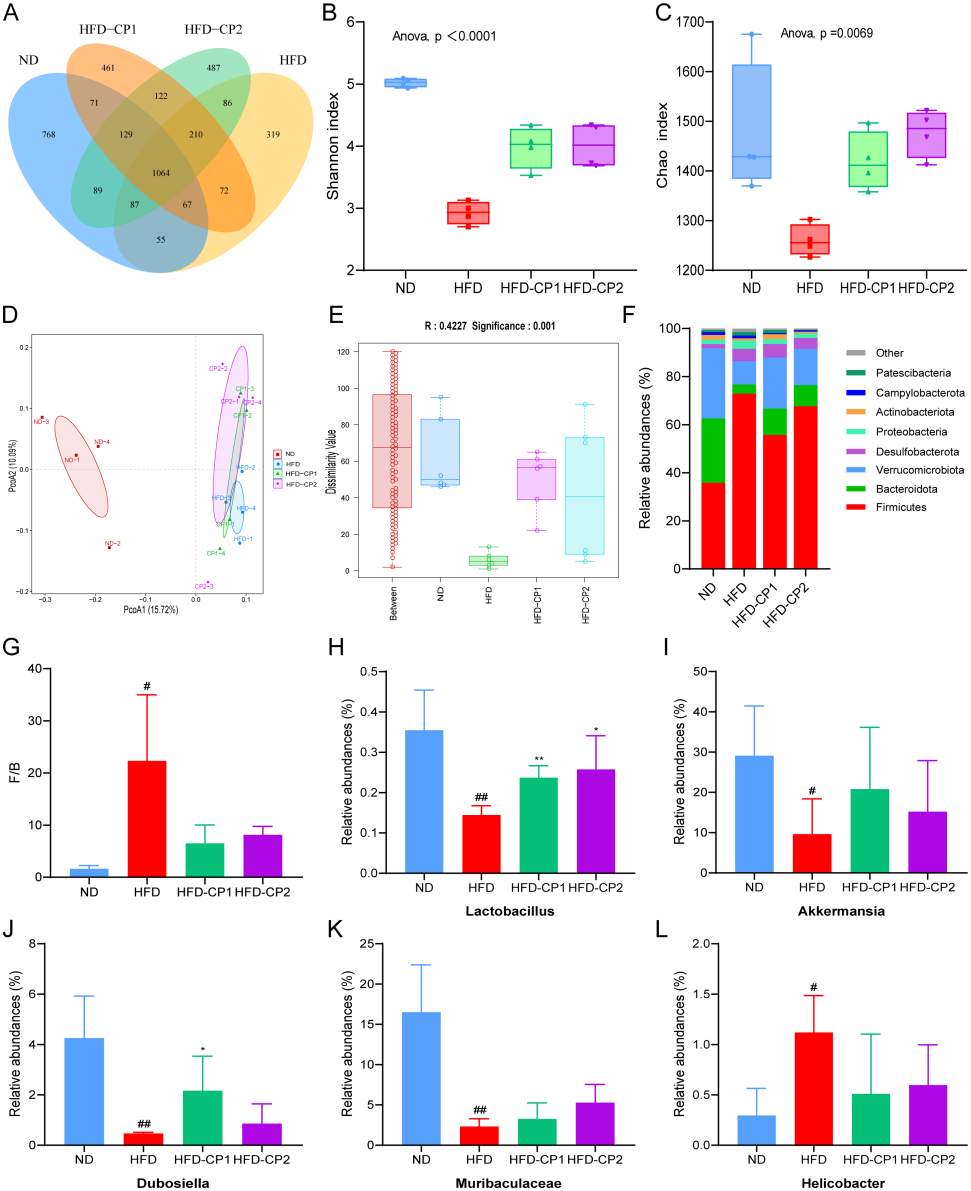
Effects of L-PPRS intervention on gut microbiota abundance and diversity in designated mouse groups. **(A)** Venn diagram. **(B)** Shannon index. **(C)** Chao index. **(D)** PCOA analysis. **(E)** Anosim analysis. **(F)** Phylum level. **(G)** The *Firmicutes*/*Bacteroidetes* ratio of intestinal microbiome in mice. **(H)** Relative abundance of the *Lactobacillus* genus. **(I)** Relative abundance of the *Akkermansia* genus. **(J)** Relative abundance of the *Dubosiella* genus. **(K)** Relative abundance of the *Muribaculaceae* genus. **(L)** Relative abundance of the *Helicobacter* genus (^#^*p* < 0.05, ^##^*p* < 0.01 vs. ND; *p < 0.05 vs. HFD).

The Diversity of species distributions were analyzed by Venn diagram and α-diversity. As shown in Figure 7A, 1,064 common Operational Taxonomic Units (OTUs) were shared in four groups. There are 768 and 319 unique OTUs in the ND group and HFD group, respectively, indicating that HFD challenge reduced the species specificity and diversity of gut microbiota. This notion was further evidenced by decreased Chao and Shannon indexes in HFD group (Figures 7B,C). Whereas, after L-PPRS intervention, unique OTUs were increased to 461 and 487 in the HFD-CP1 group and the CP2 group, respectively. Additionally, the increased Chao and Shannon indexes in HFD-CP1 and CP2 groups indicated an increased α-diversity of the gut microbiota (Figures 7B,C), revealing the recovering function of L-PPRS in the species diversity of the gut microbiota in HFD challenged mice.

Principal coordinate analysis (PCOA) (Figure 7D) and ANOSIM analysis (Figure 7E) revealed significant differences between groups. Changes in the gut microbiota at the phylum level are shown in Figure 7F. The dominant microorganisms in the intestinal flora were *Firmicutes, Bacteroidota* and *Verrucomicrobiota*, which occupied more than 88% of the total richness (Figure 7F). HFD challenge significantly increased the richness of *Firmicutes* but decreased the richness of *Bacteroidota* and *Verrucomicrobiota* (Figure 7F), and hence increased the F/B value, when compared to the ND group (Figure 7G). In contrast, the F/B value in the L-PPRS intervention groups decreased significantly. In addition, at the genus level, L-PPRS intervention not only significantly improved the abundance of *Lactobacillus* (Figure 7H), but also enhanced the abundance of other beneficial bacteria, including *Akkermansia* (Figure 7I), *Dubosiella* (Figure 7J), *Muribaculaceae* (Figure 7K). Meanwhile, the elevation of *Helicobacter* in HFD challenged mice was reversed by L-PPRS intervention (Figure 7L). Overall, these observations indicate that L-PPRS can modulate the balances of gut microbiota in HFD challenged mice.

### 3.8 Correlations between gut microbiota and obesity-related parameters

**Figure 8:**
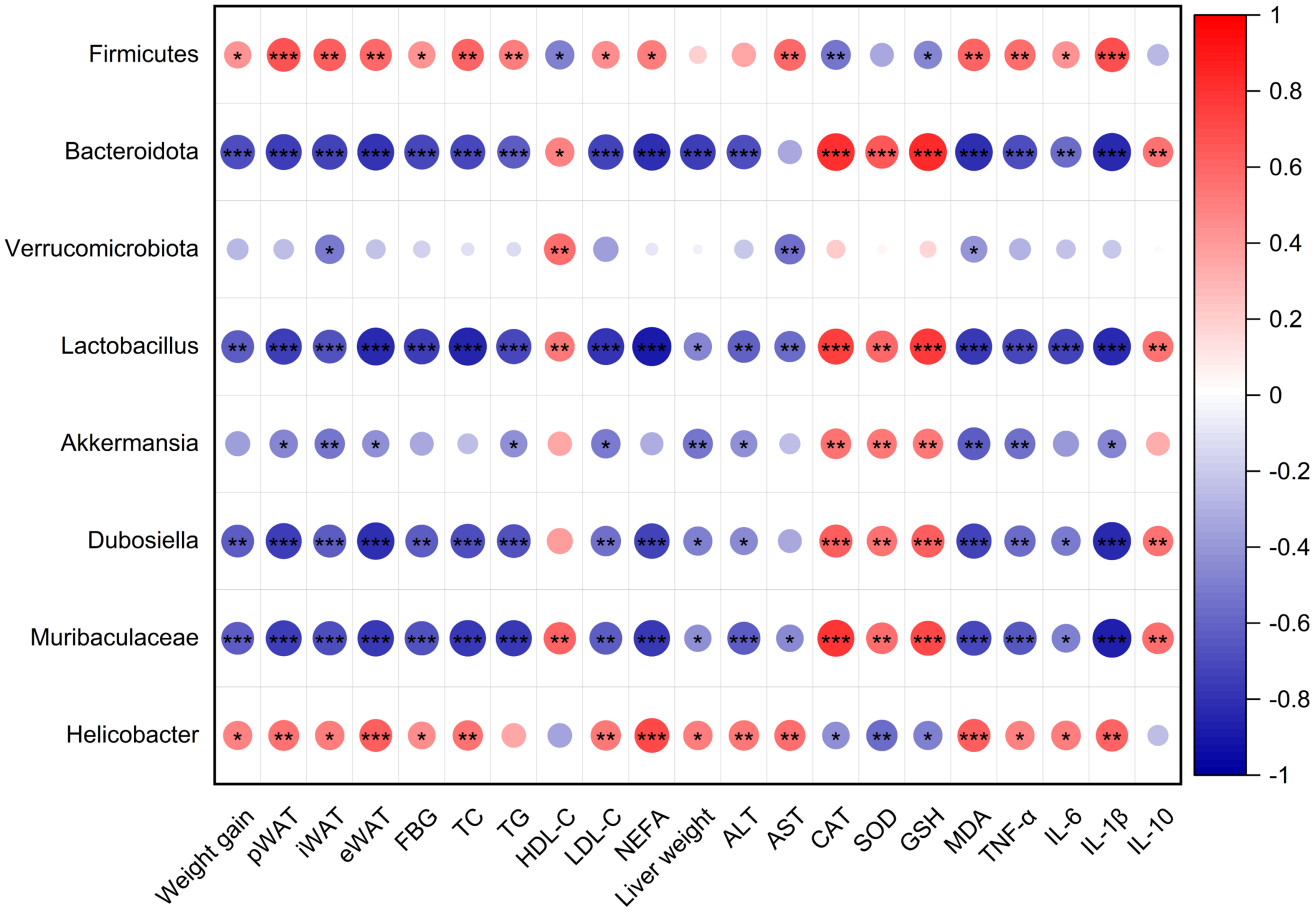
Spearman’s correlation analysis between gut microbiota and obesity-related metabolism parameters. Red dots refer to positive correlation and blue dots for negative correlation. Significant difference was marked with an asterisk (*p < 0.05, **p < 0.01, ***p < 0.001).

To further elucidate the consequences of altered gut microbiota composition, we performed the spearman correlation analysis between the gut microbiome and the obesity-related parameters, which include the weight gains in body and adipose tissues, serum lipid levels, serum inflammatory factors, live function and antioxidant capacity in livers. As shown in Figure 8, except *Verrucomicrobiota*, the weight gains in body and adipose tissues are significantly positively correlated with *Firmicutes* (at Phylum level) and *Helicobacter* (at genus level), but negatively correlated with *Bacteroidota* (at Phylum level), *Lactobacillus*, *Akkermansia*, *Dubosiella*, and *Muribaculaceae* (at genus level). There are also the same trends for the lipid levels (except HDL-C), FBG and pro-inflammatory factors in serum, liver weight, and ALT, AST and peroxides level (MDA) in the liver. In contrast, levels of serum HDL-C and anti-inflammation cytokine IL10, and the antioxidant capacity of liver exhibited the opposite correlation with these gut microbiomes. These observations indicate that gut microbiota alteration played a vital role in the alleviation of obesity and related abnormalities in mice, and L-PPRS intervention can induce the increases of *Bacteroidota*, *Lactobacillus*, *Akkermansia*, *Dubosiella*, and *Muribaculacea*, and decrease of *Firmicutes* and *Helicobacter*, contribute to this effect.

## 4 Discussion

Obesity and overweight as predisposing factors for various chronic diseases, have become important global concerns (52). Probiotics have been considered as effective treatments for obesity, as they could reverse microbial dysbiosis and regulate related metabolic disorders (53, 54). While single-strain probiotics are helpful, multi-strain probiotics could bring more profound beneficial effects due to their potential synergistic and additive benefits for health (55). It is important to note that efficacy of probiotics was both strain-specific and disease-specific (56). Mixture of two *Lactobacillus plantarum* strains was proved to be more effective in combating fat formation in a mouse model, indicating the potential application of the mixture as a dietary supplement or therapeutic agent for combating obesity (57). Another six strains-composite probiotics effectively alleviated obesity and related metabolic abnormalities in the HFD-induced obese rat model (58). The composite probiotic formulation increased the chance of colonization and survival in the gut (48). At the same time, multi-strain probiotics collected different characteristics of individual strain, and might exert their effects by strain–strain interaction, thereby might present synergy on treating diseases (49, 55). In our current study, L14, L9, LGG, and L-X-MRS-2, which are proved to be obesity-specific, are combined to a probiotic mixture L-PPRS. Our results showed that L-PPRS can effectively prevent the development of obesity and related abnormalities.

Abnormal increase in the size and number of adipocytes is the main pathological characteristic of obesity, thus inhibiting the lipid accumulation in adipocytes in the *in vitro* settings was proved to be a simple and effective way in anti-obesity research (59–61). In our current study, the anti-adipogenic effect of L14, L9, LGG, and L-X-MRS-2 and their mixture L-PPRS were evaluated in differentiated 3T3-L1 cells. Compared with the single-strain probiotics, L-PPRS decreased lipid accumulation more effectively in differentiated 3T3-L1 cells, presenting more potential on anti-obesity.

In our *in vivo* study, L-PPRS intervention was shown to reduce body weight gain and fat accumulation in the adipose tissue. Obesity is often accompanied by lipid metabolic disorders, and subsequent organ dysfunction (62, 63), and the main characteristics are abnormality in lipid components in the blood, liver, and other tissues. An increase of NEFA in the blood and liver could accelerate TG synthesis in the liver, resulted in the fat accumulation in liver, causing liver damage (64, 65). When the liver is injured, ALT and AST are released into the blood, leading to their serum level elevation (66, 67). The liver is an organ that is particularly exposed to ROS, the oxidative stress is implicated in liver injury (68). The production of ROS in the body together with the under-production of anti-oxidant mechanisms contribute to the development of obesity and related complications (69, 70). In this study, HFD led to increase of lipid levels in the serum and liver, and degeneration of fatty liver in mice. Additionally, HFD feeding also led to increases in serum ALT and AST levels, MDA levels and decreases of SOD, CAT and GSH in obese mice, indicating that liver was damaged. After L-PPRS intervention, abnormalities on lipid levels in blood and liver were significantly reversed, associated with reduced serum levels of ALT and AST. Furthermore, the redox balance of liver was improved, and liver fatty degeneration was alleviated, suggesting that the liver injury was alleviated.

Obesity was reported to be a state of low-grade chronic inflammation, and the long-term inflammation is implicated in the development of obesity and insulin resistance (19, 71, 72). In response to fat accumulation in adipose tissue, macrophages are recruited and infiltrate the adipose tissue, releasing pro-inflammatory cytokines that promote both local and systemic inflammatory responses (73). TNF-α was identified as the first pro-inflammatory adipokine (74). It can activate intracellular signaling molecules, such as JNK and IKKβ, leading to insulin resistance (75, 76). Activation of IKKβ results in the expression of nuclear factor κB (NF-κB), which drives the production of other pro-inflammatory cytokines, particularly IL-6 and IL-1β (77). IL-1β plays a critical role in adipocyte differentiation and insulin sensitivity, with its levels significantly elevated in obese individuals (78). Conversely, IL-10 expression is reduced in the adipose tissue of obese mice, and restoring IL-10 levels was shown to alleviate inflammation and improve metabolic function (79). In our current study, HFD feeding led to the increases of pro-inflammatory factors (TNF-α, IL-1β, IL-6), while the level of anti-inflammatory factor IL-10 was decreased. Concurrently, the obese mice exhibited abnormal glucose tolerance and insulin resistance. After L-PPRS intervention, the above abnormalities were effectively reversed, indicating that L-PPRS has great potential to alleviate inflammation and improve insulin sensitivity in obese mice.

Numerous studies have demonstrated that AMPK plays a pivotal role in modulating the development of obesity by regulating physiological activities including feeding, insulin sensitivity, lipid metabolism and others (80, 81). Activating AMPK pathway in adipocytes results in the inhibition of fat synthesis and enhancing lipolysis. Increased AMPK-α was shown to downregulate adipogenic transcriptional factors including C/EBPα, PPARγ, and SREBP-1c (41, 82, 83). SREBP-1c is involved in adipogenesis by activating the expression of fatty acid synthase (FAS) (84). ACC catalyzes the carboxylation of acetyl-CoA, which is the first step in fatty acid biosynthesis (85). For lipolysis, CPT-1 regulates mitochondrial fatty acids oxidation (86), while HSL are the key hydrolase in the degradation of TG and diglycerides (87). We found that L-PPRS intervention activated expression of AMPK-α, downregulated the expression of adipogenic transcriptional factors including C/EBPα, PPARγ and SREBP-1c, then decreased expression of the lipid synthesis-related genes including ACC and FAS, enhanced expression of CPT-1and HSL to promote fat breakdown, thereby regulating lipid metabolism and attenuate obesity. Further investigations are needed to confirm these transcriptional changes in protein levels.

Gut microbiota, a complex organ system, is crucial for the health. The diversity and abundance of certain bacteria may contribute to metabolic pathways resulting in obesity (88). In the gut microbiome, the *Firmicutes* and *Bacteroidetes* are the most prevalent (33). The gut microbiota of obese animals usually exhibits a higher F/B ratio, proposing this ratio as an obesity biomarker (89, 90). Our current study also confirmed this phenomenon, at the phylum level, the abundance of *Firmicutes* increased and the abundances of *Bacteroidetes* and *Verrucomicrobia* decreased in the HFD group, leading to dysbiosis and a significant increase in the F/B ratio. After L-PPRS intervention, the abundance of *Firmicutes* decreased, the abundances of *Bacteroidetes* and *Verrucomicrobia* increased, and the F/B ratio was reduced. At the genus level, L-PPRS intervention increased the abundance of *Lactobacillus*, *Akkermansia*, *Muribaculaceae*, and *Dubosiella*, and reduced the abundance of *Helicobacter*. *Akkermansia*, as the paradigm for next-generation beneficial gut microbiomes, could prevent intestinal inflammation, regulate lipid metabolism, and reduce obesity-related metabolic syndrome induced by HFD in mice (91, 92). In regard to *Muribaculaceae*, it exhibited a cross-feeding relationship with *Lactobacillus*, and produced short-chain fatty acids (93), which could alleviate systemic inflammation induced by HFD challenge (94). Extracorporeal replenishment of *Dubosiella* was reported to protect against hepatic lipid accumulation (95). Consistent with our findings, *Lactobacillus plantarum* S58 and β-glucan increased beneficial bacteria such as *Akkermansia* and *Dubosiella*, while reducing disease-related bacteria *Helicobacter* (41). Mulberry also increased *Akkermansia* and *Muribaculaceae* (96). Our experiment indicates that L-PPRS could increase the abundance of beneficial bacteria and reduce the harmful bacteria, thereby restore the diversity and richness of the gut microbiota in mice with HFD challenge.

In our current study, the HFD-CP2 group exhibited slightly better effects than the HFD-CP1 group, not only on reducing body weight gain and adipocyte size, but also on improving insulin sensitivity, normalizing serum HDL-C and LDL-C levels, and on attenuating liver damages. This might be attributed to additive effects of the long-term and early use of L-PPRS. Long-term and early intervention of L-PPRS, on the one hand, significantly promoted the expression of AMPK-α and downregulated CEBP-α related in lipid synthesis, and increased expression of lipolysis-related gene CPT-1, thereby improving lipid homeostasis. On the other hand, long-term and early intervention of L-PPRS was more effective on increasing the α-diversity of the gut microbiota and abundance of *Lactobacillus*, thereby reversing the gut microbiome dysbiosis. Therefore, probiotics treatment of obesity may be more effective if it is in early or long-term intervention.

In our current research, L-PPRS intervention alleviated obesity in HFD challenged obese mouse model, indicating its potential application in clinical studies. However, several considerations should be addressed before conducting future clinical trials. Animal studies may not fully predict human responses due to potential individual differences in age, diet, genetics, and other environmental factors. Caloric intake should be controlled or reported to isolate the effects of L-PPRS. Additionally, drug dosages used in animal experiments need to be appropriately adjusted based on body surface area or weight to ensure safe and effective translation to humans (97).

Our study demonstrated that L-PPRS intervention effectively alleviated obesity induced by HFD challenge. L-PPRS intervention not only significantly suppressed body weight gain and fat accumulation in HFD challenged mice but also improved glucose tolerance, insulin sensitivity and attenuated serum lipid levels. Furthermore, L-PPRS intervention improved liver function, restored the redox balance of liver, and attenuated inflammatory responses in HFD challenged mice. In addition, L-PPRS modulated the expression of lipid-metabolic genes. What’s more, L-PPRS effectively restored the dysbiosis of the gut microbiota caused by HFD challenge, reduced the F/B ratio, and increased the abundance of beneficial bacteria, thereby attenuated obesity. In conclusion, L-PPRS could effectively prevent the development of obesity and related abnormalities, and the long-term or early supplementation of L-PPRS would provide a more profound benefit.

## Data Availability

The original contributions presented in the study are included in the article/Supplementary material, further inquiries can be directed to the corresponding authors.
