# Supplementary material for: Supplementation of mixed Lactobacillus alleviates metabolic impairment, inflammation, and dysbiosis of the gut microbiota in an obese mouse model
Source: Front Nutr. 2025 Mar 26;12:1554996. doi: 10.3389/fnut.2025.1554996 (PMC11978641; doi:10.3389/fnut.2025.1554996)
Supplement: Supplementary file 1 [file Data_Sheet_1.pdf]

Supplementary Material

1 Supplementary Figures and Tables

1.1 Supplementary Figures

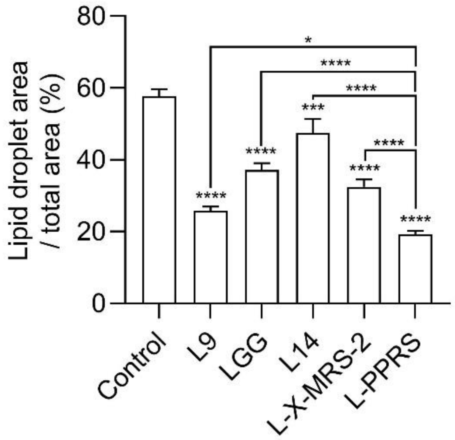

Supplementary Figure 1. The ratio of the lipid droplet area to the total area. (* p <0.05, ** p<0.01, *** p<0.001, **** p < 0.0001)

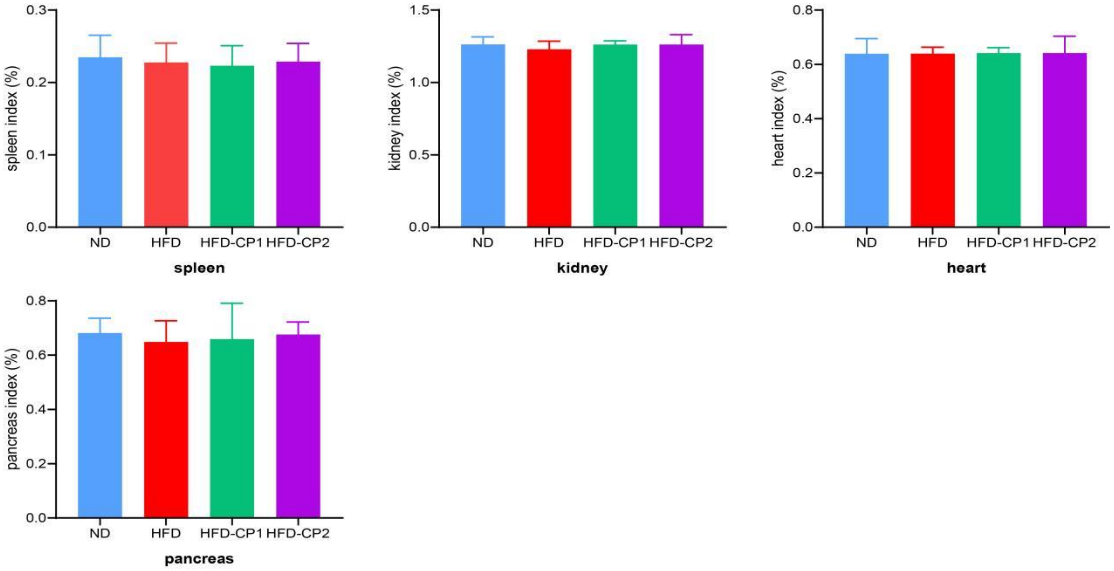

Supplementary Figure 2. The organ index of the spleen, kidney, heart and pancreas.

1.2 Supplementary Tables

Primers	Nucleotide sequence of primers (5’-3’)
β-actin	F: GGCTATGCTCTCCCTCACG<br>R: CGCTCGGTCAGGATCTTCAT
AMPK-α	F: CCCTGTGTATGTGGCTCTG<br>R: GTGGGTGAACCTCTGCTT
ACC	F: GGGAATACCTGTGGGAGTAGT<br>R: GCTGGATTATCTTGGCTTCA
FAS	F: TGCTTGCTGGCTCACAGTTAAGAG<br>R: TTCACGAACCCGCCTCCTCAG
PPAR-γ	F: TGTTCGCCAAGGTGCTCCAG<br>R: AAGGCTCATGTCTGTCTCTGTCTTC
SREBP-1C	F: CCACCCTGTAGGTCACCGTTTC<br>R: CGCTCGCTCTAGGAGATGTTCAC
CPT-1	F: GGGCAGATTCTTCCGTGTAGGG<br>R: CAGTCAGAGCAGCTAGGTGTTCC
CEBP-α	F: GTAACCTTGTGCCTTGGATACT<br>R: GGAAGCAGGAATCCTCCAAATA
HSL	F: CTCACAGTTACCATCTCACCTC<br>R: GATTTTGCCAGGCTGTTGAGTA

Supplementary Table 1. Primer sequences for RT-PCR on target genes that are related to lipid metabolism.
